# Evolution of Ceftriaxone Resistance of Penicillin-Binding Proteins 2 Revealed by Molecular Modeling

**DOI:** 10.3390/ijms24010176

**Published:** 2022-12-22

**Authors:** Alexandra V. Krivitskaya, Maria G. Khrenova

**Affiliations:** 1 Bach Institute of Biochemistry, Federal Research Centre “Fundamentals of Biotechnology” of the Russian Academy of Sciences, 119071 Moscow, Russia; 2 Department of Chemistry, Interdisciplinary Scientific and Educational School of Moscow University “Brain, Cognitive Systems, Artificial Intelligence”, Lomonosov Moscow State University, 119991 Moscow, Russia

**Keywords:** bacterial antibiotic resistance, QM/MM, molecular dynamics, ceftriaxone, reaction mechanism

## Abstract

Penicillin-binding proteins 2 (PBP2) are critically important enzymes in the formation of the bacterial cell wall. Inhibition of PBP2 is utilized in the treatment of various diseases, including gonorrhea. Ceftriaxone is the only drug used to treat gonorrhea currently, and recent growth in PBP2 resistance to this antibiotic is a serious threat to human health. Our study reveals mechanistic aspects of the inhibition reaction of PBP2 from the wild-type FA19 strain and mutant 35/02 and H041 strains of *Neisseria Gonorrhoeae* by ceftriaxone. QM(PBE0-D3/6-31G**)/MM MD simulations show that the reaction mechanism for the wild-type PBP2 consists of three elementary steps including nucleophilic attack, C–N bond cleavage in the β-lactam ring and elimination of the leaving group in ceftriaxone. In PBP2 from the mutant strains, the second and third steps occur simultaneously. For all considered systems, the acylation rate is determined by the energy barrier of the first step that increases in the order of PBP2 from FA19, 35/02 and H041 strains. Dynamic behavior of ES complexes is analyzed using geometry and electron density features including Fukui electrophilicity index and Laplacian of electron density maps. It reveals that more efficient activation of the carbonyl group of the antibiotic leads to the lower energy barrier of nucleophilic attack and larger stabilization of the first reaction intermediate. Dynamical network analysis of MD trajectories explains the differences in ceftriaxone binding affinity: in PBP2 from the wild-type strain, the β_3_-β_4_ loop conformation facilitates substrate binding, whereas in PBP2 from the mutant strains, it exists in the conformation that is unfavorable for complex formation. Thus, we clarify that the experimentally observed decrease in the second-order rate constant of acylation (*k*_2_/*K*_S_) in PBP2 from the mutant strains is due to both a decrease in the acylation rate constant *k*_2_ and an increase in the dissociation constant *K*_S_.

## 1. Introduction

*Neisseria gonorrhoeae* is a Gram-negative diplococci bacterium. It causes sexually transmitted infections, disseminated gonococcemia, septic arthritis, and gonococcal ophthalmia neonatorum. It took these bacteria 40 years to develop mechanisms of resistance to penicillin G. Further growth of resistance was more rapid [1,2]. In 2007, a list of recommended drugs for the treatment of infections caused by these bacteria was presented. However, after only three years, the only therapy that remained was ceftriaxone together with azithromycin. Emergence of resistance to azithromycin [3] resulted in ceftriaxone monotherapy as a major way of treatment in some countries. Still, failure of this therapy is already reported. Currently, there are several new compounds being clinically studied for treatment [4,5,6,7]. One of them, zoliflodacin, is in phase 3 trials and is considered as the most promising compound to replace ceftriaxone in case of further grown of *Neisseria gonorrhoeae* resistance.

There is a number of determinants associated with the mutation of genes responsible for emerging multiple resistance [8]. They occur in gene *penA*, which encodes an essential transpeptidase involved in cell division, penicillin-binding protein 2 (PBP2); gene *mtrR*, which increases the expression of the MtrC-MtrD-MtrE efflux pump [9,10]; gene *penB*, encoding the constriction loop of the PorB1b porin [11,12]; and gene *ponA* encoding another essential PBP in the gonococcus, PBP1 [13]. Resistance to antibiotics of the cephalosporin group is more associated with the occurrence of mutations in gene *penA*, which encodes PBP2 enzyme [14,15].

In general, PBPs are enzymes involved in the final stages of peptidoglycan synthesis in bacteria. PBPs are grouped either into three main classes according to their functions, A, B, and C, or two classes according to their molecular weight with high (HMM) and low (LMM) molecular masses [16]. Class A and B PBPs are transpeptidases that catalyze the formation of peptide cross-links between adjacent glycan strands of peptidoglycan. Class A also contains a N-terminal transglycosylase domain that polymerizes glycan strands, whereas class B owns only transpeptidase activity. Class C catalyzes carboxypeptidase activity in vitro and may modulate the degree of cross-linking in peptidoglycan [16,17].

*N. gonorrhoeae* have four PBPs [18]: two high molecular mass transpeptidases, class A PBP1 and class B PBP2, and two low molecular mass, class C PBP3 and PBP4. PBP3 and PBP4 do not essentially affect the process of cell growth, while PBP1 and PBP2 are vitally important [19]. PBP2 is supposed to be the major target for cephalosporin, as it is inhibited by lower antibiotic concentrations compared with PBP1 [20]. Therefore, we consider transpeptidase PBP2 from *N. gonorrhoeae* from the penicillin-susceptible strain, FA19, and penicillin-resistant strains, 35/02 and H041.

PBP2 from the 35/02 strain (PBP2^35/02^ in the following text) is the extended-spectrum cephalosporin (ESC)-susceptible strain. This enzyme contains 57 amino acid substitutions compared with PBP2 from the FA19 strain (PBP2^FA19^). Three of them, I312M, V316T and G545S, are mainly responsible for the resistance to ESCs [21]. The second-order rate constant of acylation (*k*_2_/*K*_S_) of PBP2^35/02^ by ceftriaxone is 150-fold lower compared with PBP2^FA19^ [22]. PBP2 from the H041 strain (PBP2^H041^) contains 61 amino acid mutations compared with PBP2^FA19^ [23], and 13 of them differ from that in PBP2^35/02^. Three of the mutations, A311V, V316P, and T483S, are shown to be mostly responsible for the resistance to ceftriaxone and cefixime [14]. The *k*_2_/*K*_S_ for PBP2^H041^ acylation by ceftriaxone is 2300-fold lower compared with PBP2^FA19^ [22]_._

The second-order rate constants of PBP2 acylation by ceftriaxone, *k*_2_/*K*_S_, is a ratio of the rate constant *k*_2_ of the covalent PBP2–ceftriaxone complex formation and a thermodynamic ceftriaxone–PBP2 dissociation constant, *K*_S_ [14,21]. The individual contribution of these two constants was indirectly determined only for PBP2^FA19^ in an isothermal titration calorimetry study. The dissociation constant was measured for the S310A variant of PBP2^FA19^ that lacks a catalytic serine residue, supposing that the dissociation constant should not be affected by this substitution [22]. The *k*_2_ value was evaluated from the second-order rate constants and the *K*_s_ value for PBP2^FA19^-S310A. For PBP2 from the mutant strains, it was not possible to determine *K*_S_ due to the weak binding of ceftriaxone. Recent NMR studies reveal that the increase in antibiotic resistance due to the increase in the *K*_s_ value is associated with changes of β_3_-β_4_ loop conformation [24]. Resistance to ceftriaxone increases with the destabilization of the inward β_3_-β_4_ loop conformation and stabilization of the its extended conformation [24]. Therefore, it remains unclear whether changes in *k*_2_/*K*_S_ of PBP2 from the FA19 and mutant strains are governed by the changes in the acylation rate constant or dissociation constant, or by these parameters.

In this work, we comprehensively study molecular mechanisms of interactions between PBP2 from FA19, 35/02 and H041 strains and antibiotic ceftriaxone using modern methods of computational chemistry. We utilize QM(PBE0-D3/6-31G**)/MM MD simulations to obtain Gibbs energy profiles of all elementary steps of acyl–enzyme complex formation. We perform electron density analysis of different states in ES complexes to explain how efficiency of substrate activation by enzyme affects the following nucleophilic attack. In addition, we perform dynamical network analysis to compare the antibiotic binding affinity by PBP2 from different strains.

## 2. Results

### 2.1. Ceftriaxone Activation in the Active Site of Enzyme and Nucleophilic Attack

**Figure 1:**
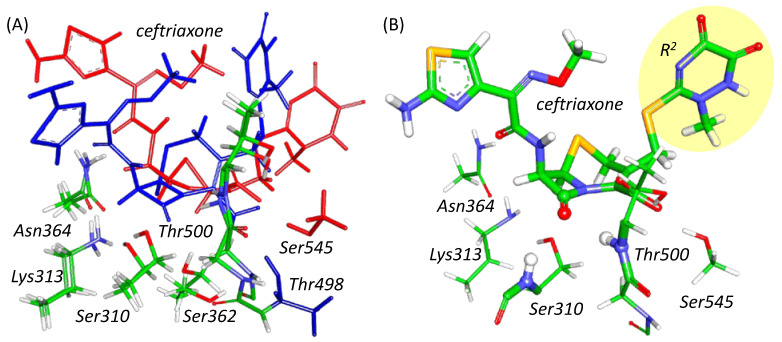
(**A**) Superposition of active site fragments of PBP2^FA19^ (blue) and PBP2^H041^ (red) with bound ceftriaxone. Alignment is performed over nitrogen and carbon atoms of main chains of Ser310, Lys313 and Thr500. (**B**) The QM part of PBP2^H041^ at the ES configuration; the leaving group of ceftriaxone (R^2^) is highlighted by a yellow ellipse. The carbonyl group of the β-lactam ring and NH fragments of Thr500 and Ser310 residues forming the oxyanion hole are represented as balls and thick sticks. Ceftriaxone and other amino acid residues are depicted with thick and thin sticks, respectively. Here and in the following figures, carbon is green, oxygen is red, nitrogen is blue, hydrogen is white, and sulfur is yellow.

PBP2 is a serine hydrolase that shares common features with the enzymes of this type, EC 3. The active site carries a nucleophilic moiety that initiates acylation and an oxyanion hole that activates the substrate and stabilizes the tetrahedral intermediate [25,26,27]. In all considered PBP2 variants, the oxyanion hole is formed by NH groups of the main chains of Ser310 and Thr500 (Figure 1B). These interactions of the substrate with the oxyanion hole result in polarization of the carbonyl group of the antibiotic and its activation as an electrophile. A Ser310 residue serves as a nucleophile, and the nearby Lys313 residue acts as a proton acceptor during the reaction (Figure 1). Still, the active site of PBP2^FA19^ differs from PBP2 from the mutant strains, which might be responsible for the *k*_2_/*K*_s_ changes. The carboxyl group of ceftriaxone, which is conservative for β-lactam antibiotics, interacts with Thr498 and Ser362 residues in PBP2^FA19^ (Figure 1A). Both mutant PBP2 have G545S amino acid substitution that alters hydrogen bond partners of ceftriaxone carboxylate. In PBP2 from the mutant strains, Ser545 interacts with this group (Figure 1A). This interaction shifts the position of the substrate closer to the exit from the binding pocket in PBP2 from the mutant strains. This influences the following reaction as shown below.

The efficiency of substrate activation in the enzyme-substrate complex can be quantified from molecular dynamics simulation with QM/MM potentials in different ways, including simple geometry criteria and more complicated electron-density-based descriptors. A detailed analysis of the geometry features in the active sites of the ES complexes of ceftriaxone with PBP2 from different strains demonstrates considerable variations of hydrogen bond distance between the NH fragments of Ser310 and the carbonyl oxygen atom of the substrate [28]. Practically, in PBP2^FA19^, the oxyanion hole is formed by two hydrogen bonds, and in PBP2 from the mutant strains, only one hydrogen bond with Thr500 is formed.

**Figure 2:**
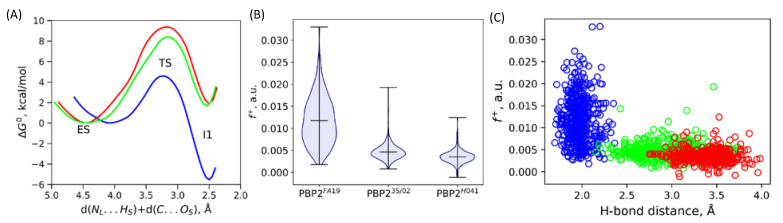
(**A**) Gibbs energy profiles of the first reaction step; a nucleophilic attack of PBP2 acylation by ceftriaxone. (**B**) Violin plots for the distributions of Fukui electrophilicity indices of the carbonyl carbon atom of the substrate, *f* ^+^, in ES state. (**C**) Scatter plots of distances of a longer hydrogen bond in the oxyanion hole at each QM/MM MD frame versus Fukui index in the corresponding frame. Color code for panels A,C: PBP2^FA19^–blue, PBP2^35/02^–green and PBP2^H041^–red.

Here, we deepen the understanding of substrate activation by the enzyme, focusing on electron-density-based descriptors. We evaluate the Fukui electrophilicity index, *f* ^+^, of the carbonyl carbon atom of ceftriaxone and Laplacian of electron density maps calculated in the plain of the carbonyl group of the substrate and a nucleophilic oxygen atom for sets of frames for each model system (Figure 2B).

First, we utilize a binary classifier, Laplacian of electron density, to evaluate the fraction of the reactive species that are characterized by the presence of the electron density depletion area on the carbonyl carbon atom in the direction of the nucleophilic attack [29,30,31]. The substrate activation is more efficient in PBP2^FA19^ and equals 33%, whereas it decreases to 16% and 12% for PBP2^35/02^ and PBP2^H041^, respectively.

Figure 2B shows the violin plots of Fukui electrophilicity indices for ceftriaxone carbonyl carbon atom for PBP2 from three considered strains. For PBP2^FA19^-containing system, *f*^+^ has a wider distribution with the larger average value compared with the other two PBP2. It is characterized by the 0.0117 ± 0.0053 a.u. mean value and standard deviation, whereas for PBP2^35/02^ and PBP2^H041^, these values are 0.0047 ± 0.0018 and 0.0035 ± 0.0014 a.u., respectively. Larger Fukui atomic electrophilicity indices discriminate atoms with more pronounced electrophilic properties. Therefore, we can conclude that in PBP2^FA19^ substrate, activation by the enzyme is considerably more pronounced. Thus, utilization of both spatially distributed Laplacian of electron density descriptor and atomic Fukui indices derives the consistent result that the substrate activation efficiency decreases in PBP2 from different strains in the following order: FA19 > 35/02 > H041.

We compare Gibbs energy profiles of the first step of the reaction to understand which particular features of the following reaction are affected by the efficiency of the substrate activation (Figure 2A). The reaction coordinate at this step is a sum of distance between the proton transferring from the Ser310 residue side chain to the Lys313 residue side chain, d(N_L_…H_S_), and a distance of the nucleophilic attack, d(C…O_S_). The energy profile for PBP2^FA19^ considerably differs from those for PBP2 from the mutant strains. The first intermediate is stabilized relative to the ES complex only in PBP2^FA19^. For PBP2 from both mutant strains, 2 kcal/mol destabilization is observed. The energy barrier is much lower for the system with PBP2^FA19^ (4.6 kcal/mol), and it increases to PBP2^35/02^ (8.4 kcal/mol) and PBP2^H041^ (9.4 kcal/mol) in accordance with the decrease in the experimental *k*_2_/*K*_S_ value. In addition, an important feature is the reaction coordinate at the ES minimum; this equals to 4.1 Å in PBP2^FA19^ and around 4.5 Å in PBP2 from the mutant strains. This indicates that in the wild-type PBP2, the ES complex is tighter, and this contributes to efficient substrate activation.

We analyze whether Fukui atomic electrophilicity indices depend on individual geometry parameters or are affected by their combination. We found no strong correlations between the individual interatomic distances and *f* ^+^. Figure 2C depicts the scatter plot of a longer distance among two hydrogen bonds forming an oxyanion hole as a geometry parameter and Fukui index. The distributions of the distance of the longer hydrogen bond in the oxyanion hole practically do not overlap for PBP2 from the wild-type and mutant strains. Those are quantified by 1.97 ± 0.12, 2.92 ± 0.30 and 3.39 ± 0.22 Å for PBP2^FA19^, PBP2^35/02^ and PBP2^H041^, respectively. We observe that the absence of a second hydrogen bond in the oxyanion hole almost absolutely eliminated states with high Fukui electrophilicity indices on the carbonyl carbon atom of the substrate as seen for PBP2 from the mutant strains (Figure 2B,C). Still, the formation of two hydrogen bonds in the oxyanion hole does not guarantee efficient substrate activation, as there is no correlation between the *f* ^+^ and this interatomic distance for PBP2^FA19^.

To understand whether the geometry parameters together determine the extent activation of the substrate, we applied multiple linear regression analysis and random forest machine learning algorithms for Fukui indices and corresponding parameters for PBP2^FA19^-containing system. From multiple linear regression, we obtain the following relation

*f* ^+^ = 0.0141·d(C…OS) − 0.0059·d(O…HSer) − 0.0039·d(O…H_Thr_) − 0.0039

This can estimate *f* ^+^ with the mean absolute value of 0.004 a.u. The distance of the nucleophilic attack comes with the positive coefficient. Distances of nucleophilic attack are relatively short in this system in different MD frames, and the influence is not obvious. For both hydrogen bond lengths, we find expected coefficients with negative signs; larger hydrogen bond distances decrease *f* ^+^ values. The coefficient for the d(O…H_Ser_) has almost a twice larger absolute value than for the d(O…H_Thr_). This is expected, as d(O…H_Ser_) is usually larger than d(O…H_Thr_) for each particular MD frame [28], and its further elongation should have a more pronounced negative effect. The multiple linear regression demonstrates a relatively large mean absolute error of 0.004 a.u., indicating that the relation is far from linear. Therefore, we tried to obtain an implicit interrelation using a random forest approach. This allowed us to decrease the mean absolute error to 0.001 a.u. and estimate the importance of each geometry parameter. We found that the importance of all parameters is similar and ranges between 0.31 and 0.37. Utilization of only one geometry parameter in the random forest model leads to an increase in the mean absolute error to 0.002 a.u. for both distance of nucleophilic attack and hydrogen bond distances in the oxyanion hole. This is in line with the observations for the cysteine protease M^pro^ from the SARS-CoV-2 virus such that both nucleophilic attack distance and oxyanion hole features should be considered [29].

### 2.2. Mechanism of Acyl–Enzyme Complex Formation in PBP2 from FA19 Strain

**Figure 3:**
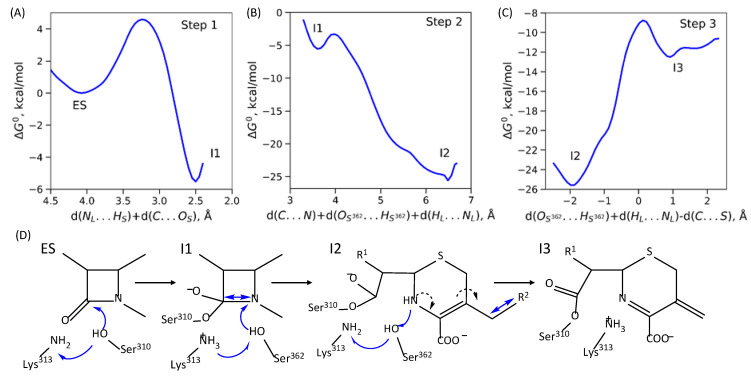
(**A**–**C**) Gibbs energy profiles of the elementary steps of acylation reaction in PBP2^FA19^–ceftriaxone complex; all energy values are relative to the ES. (**D**) Molecular mechanism of this reaction; plain blue arrows are reaction coordinates; dashed black arrows depict redistribution of electron pairs.

The pathway from the enzyme–substrate complex to the acyl-enzyme consists of three elementary steps in the case of PBP2^FA19^. The first step is a concerted process of proton transfer from the oxygen atom of the side chain of Ser310 residue to the side chain amino group of the Lys313 residue and formation of the covalent bond between the carbonyl carbon atom of ceftriaxone and an oxygen atom of the Ser310 side chain. The ES minimum on the Gibbs energy profile corresponds to the reaction coordinate 4.1 Å. When analyzing contributions from the individual interatomic distances, d(H_S_…N_L_) is around 1.6 Å and the nucleophilic attack distance, d(O_S_…C), is around 2.5 Å. The reaction coordinate decreases to 2.5 Å in the first reaction intermediate, I1. The I1 is 5.5 kcal/mol stabilized relative to the ES (Figure 3A). The second step of the reaction is the cleavage of the β-lactam ring and the protonation of the nitrogen atom of the β-lactam ring. Here, the reaction coordinate is also complex being the sum of three distances: d(C…N) corresponds to ring cleavage, d(H_L_…N_L_) is the proton transfer from a nitrogen atom of Lys313 and d(H_S362_…O_S362_) is the proton transfer from an oxygen atom of Ser362. The Gibbs energy barrier of the second elementary step is 2.3 kcal/mol, and I2 is stabilized by 20 kcal/mol relative to the I1 (Figure 3B). The third elementary step is cleavage of the covalent bond between the leaving group of ceftriaxone, R^2^, and the rest of ceftriaxone and proton rearrangements: the Ser362 proton returns to the amino group of Lys313, and the proton from the nitrogen of ceftriaxone returns to Ser362. Eliminating the leaving group provokes a redistribution of double bonds in the π-conjugated fragment of the substrate: a double bond between nitrogen and carbon of the thiazole ring is formed (Figure 3C). The energy barrier value of the third step is 17 kcal/mol.

### 2.3. Mechanisms of Acyl–Enzyme Complex Formation in PBP2 from 35/02 and H041 Strains

**Figure 4:**
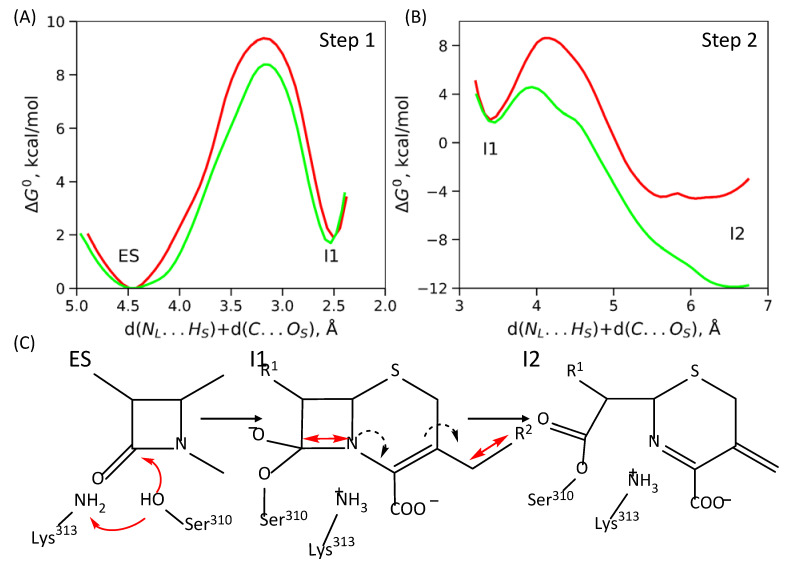
(**A**,**B**) Gibbs energy profiles of the elementary steps of ceftriaxone acylation in the active site of PBP2^35/02^ (green) and PBP2^H041^ (red); all energy values are relative to the ES. (**C**) Molecular mechanism of this reaction; plain red arrows are reaction coordinates; dashed black arrows depict redistribution of electron pairs.

As mentioned in Section 2.1 (Figure 1A), in PBP2 from the mutant strains, the substrate bound to the active site is a different way that reflects the reaction mechanism as shown below. The overall acylation reaction occurs in two elementary steps in PBP2 from the mutant strains compared with three steps in PBP2 from the FA19 strain. The second step comprises simultaneous C-N bond cleavage of the β-lactam ring and elimination of the leaving group, R^2^, of the antibiotic. The reaction coordinate at this step is the sum of the distances d(C…N) and d(C…S). The energy barrier of the second elementary reaction step is 2.8 and 6.7 kcal/mol for PBP2^35/02^ and PBP2^H041^, respectively (Figure 4B). The overall acylation reaction leads to the stabilization of the I2 state by 12 and 4 kcal/mol for PBP2^35/02^ and PBP2^H041^, respectively (Figure 4B).

### 2.4. Dynamical Network Analysis of β_3_-β_4_ Loop

**Figure 5:**
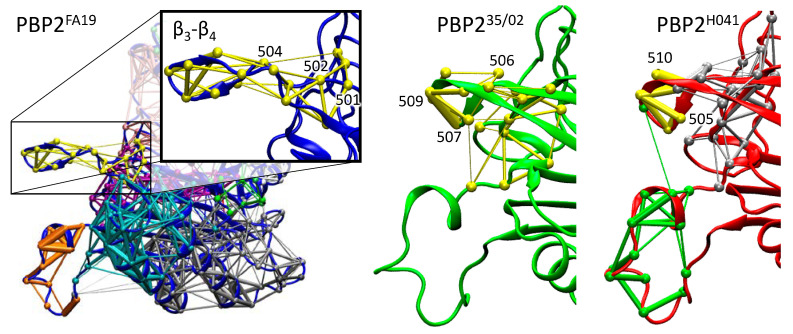
Communities obtained in dynamical network analysis of 500 ns trajectories of PBP2^FA19^ (blue), PBP2^35/02^ (green) and PBP2^H041^ (red); the loop β_3_-β_4_ is highlighted. Edges of communities are shown in sticks, and nodes are shown as balls. Edges and nodes from the same community are colored same.

Molecular dynamic trajectories with a total length of 500 ns for each system were simulated to analyze conformational diversity introduced by amino acid substitutions. Dynamical network analysis was performed to identify communities, i.e., structural regions of the protein characterized by correlated motions. The method identified 12 communities in PBP2^FA19^ and PBP2^35/02^ and 11 communities in PBP2^H041^. Particular attention was paid on the β_3_-β_4_ loop that is supposed to be responsible for the stabilization of the noncovalent PBP2–ceftriaxone complex upon binding [24]. In PBP2^FA19^, the entire loop containing residues 501 to 515 is assigned to a separate community and shares common edges between the base of the loop (residues 501, 502, and 504) and the rest of the protein (Figure 5). In PBP2^35/02^, residues 502 to 512 constitute a single community, and this community has six common edges with the protein core, including a common edge with a loop tip (residue 506). This shows that the movement of the β_3_-β_4_ loop relative to the rest of the protein is limited. Moreover, at the tip of the β_3_-β_4_ loop, there is a thick edge between residues 507 and 509, which indicates that the movement in the loop is highly correlated; the tip does not open and rotate as in PBP2^FA19^, but exhibits flat and stretched conformation. The β_3_-β_4_ loop behavior is similar in PBP2^H041^ and PBP2^35/02^. In PBP2^H041^, the difference from PBP2^FA19^ is more pronounced, as a separate community is formed except in the 505 to 510 residues, and the edges are thicker, indicating a more pronounced correlation in this region (Figure 5, PBP2^H041^, silver). In addition, the middle of the loop is assigned to another community, common with the protein core, and the tip of the loop has a common edge with the loop located under β_3_-β_4_ (Figure 5, PBP2^H041^, green). Thus, dynamical network analysis reveals that the β_3_-β_4_ loop moves independently from the rest of the protein and demonstrates more twisted conformations in the in PBP2^FA19^ in contrast to that from PBP2 from the mutant strains. Combining these data with experimental studies reposted in Ref [24], we can conclude that PBP2^FA19^ should have higher affinity to ceftriaxone.

## 3. Discussion

According to the experimental studies, interactions of ceftriaxone and PBP2 are described by a three-step model:

$$E+S\overset{K_{S}}{↔}E\cdot S\overset{k_{2}}{\to }E-S\prime \overset{k_{3}}{\to }E+P$$

where *E* is the enzyme, *S* is an antibiotic, *E·S* is a noncovalent enzyme-substrate complex, *E − S’* is a covalent acyl–enzyme complex, and *P* is a hydrolyzed antibiotic. *K_S_* is a dissociation constant and characterizes the noncovalent complex formation, *k*_2_ and *k*_3_ are the rate constants of the acylation and deacylation steps, respectively [32]. According to the complex scheme of the experimental determination of *k*_2_*/K_S_* values for ceftriaxone reaction with PBP2 [32], *k*_2_ is referred to as the formation of the covalent complex between PBP2 and ceftriaxone. In the reaction mechanisms proposed here, the intermediates I1, I2 and I3 are in the system with PBP2^FA19^ and I1, I2 are in the systems with PBP2 from the mutant strains, as a covalent adduct is already formed after the first elementary step. In other words, for the experimentally proposed mechanism, there is no particular composition of *E-S´* with respect to the theoretically obtained intermediates. In PBP2^FA19^, all intermediates starting with I1 are stabilized relative to the ES; therefore, forward reactions occur with a higher rate than backward ones, and the first forward step should be considered when compared with the experimental *k*_2_ value. For PBP2 from the mutant strains, I1 is destabilized relative to the ES; still, the following step resulting in the stabilized I2 state is characterized by a lower energy barrier. Therefore, for PBP2^35/02^ and PBP2^H041^, the first forward step determines the overall process of acyl-enzyme complex formation. The energy barrier of the first forward step increases in the series of PBP2^FA19^, PBP2^35/02^ and PBP2^H041^ enzymes, indicating that the rate constant decreases in the same order.

Analysis of crystal structures further supports the proposed mechanisms and their differences between PBP2 from FA19 and mutant strains. For FA19, the crystal structure PDB ID 6P54 [33] presents a dimer, and in one monomer acyl-enzyme complex, there exists the leaving group R^2^ (I2 in the mechanism on Figure 3), and in the other monomer, no R^2^ fragment is observed (I3 in the mechanism on Figure 3). This is because the I2 state is stabilized and accumulates rapidly during the reaction. Then, it slowly proceeds to the I3 state without an R^2^ fragment. I3 is less stable than I2; after the cleavage of the bond between the R^2^ and the rest of ceftriaxone, leaving group dissociates to the solution, and thus, I2 to I3 transition may occur only in the forward direction. In contrast, in the PBP2^H041^ crystal structure PDB ID 6VBD [22], the acyl–enzyme complex exists without a leaving group that is in line with the proposed mechanism (Figure 4) with a direct formation of the stabilized state I2.

Explicit estimates of dissociation constants usually lead to large errors; therefore, we relied on experimental studies of the conformational diversity of PBP2 and performed molecular dynamic simulations. This allowed us to indirectly compare binding affinities of three considered PBP2. In long MD trajectories, we found that the behavior of the β_3_-β_4_ loop differs in the considered systems. In wild-type PBP2, it mostly exists in conformation that facilitates the increase in binding affinity, whereas PBP2 from both mutant strains demonstrate conformations that are not favorable for efficient binding.

Thus, experimentally observed decrease of the *k*_2_*/K_S_* values in PBP2 from the mutant strains relative to the wild-type PBP2 is due to both decrease in the *k*_2_ value and increase in the dissociation constant.

## 4. Methods

The complex of PBP2^FA19^ with ceftriaxone was obtained from the crystal structure PDB ID 6P54 [33] with a resolution of 1.8 Å, and the complex of PBP2^H041^ with ceftriaxone was obtained from the crystal structure PDB ID 6VBD [22] with a resolution of 1.8 Å. The crystal structure PDB ID 6VBL [22] of PBP2^35/02^ *apo*-form with a resolution of 1.9 Å was used as the initial approximation for the coordinates. For all structures, amino acid residues 282–298 are not resolved in X-ray structures and therefore were reconstructed according to the primary amino acid sequences from UniProt. Ceftriaxone substrate was introduced in the active site of the *apo*-form of the crystal structure of PBP2^35/02^ similarly to its complex with PBP2^H041^. Hydrogen atoms were added using the Reduce program [34] in such a way that the protonated forms of the amino acids with ionogenic groups corresponded to the neutral pH, except for the catalytic residue Lys313, which remained in the neutral form, being a proton acceptor from the catalytic residue Ser310 during the nucleophilic attack. Thus obtained full atomic models of the complexes were solvated by water molecules to rectangular boxes in which the distance from any atom of a protein to the cell boundaries was at least 12 Å. Then, chlorine anions were added to the system to neutralize the total charge of the cell. Complexes were equilibrated for 20 ns using the CHARMM36 [35,36] force field for the protein, TIP3P [37] for water molecules, and CGenFF [38] for ceftriaxone. Classical molecular dynamics (MD) simulations were performed at T = 300 K and p = 1 atm with the 1 fs integration time step. Calculations were performed using the NAMD program package [39]. The root mean-square deviation (RMSD) values along the obtained trajectories show that this length is enough for the complete relaxation of the system. Then, the representative frames from the last 2 ns of the MD runs were used to prepare the QM/MM models for calculation of reaction mechanisms.

The quantum subsystem includes the substrate molecule, catalytic residues Lys313 and Ser310, amino acid residues forming the oxyanion hole, interacting with substrate or catalytic residues, and water molecules (Figure 1B). For PBP2^FA19^, they are Ser362, Asn364, Lys497, Thr498, and Thr500, and for PBP2 from mutated strains, they are Ser362, Asn364, Thr500 and Ser545. The quantum mechanical moiety was then described at density functional theory level in the Kohn–Sham variant with PBE0 hybrid functional [40] and D3 dispersion correction [41] in the 6-31G** basis set. The MD calculations were performed for 5 ps with the QM/MM potentials for each enzyme-substrate complex in the NAMD program package combined with the TeraChem [42] complex integrated for quantum chemical calculations [43]. The cutoff distance for point charges of the MM subsystem contributing to the QM Hamiltonian was 12 Å. PBE0 function is utilized, as it demonstrates consistent results for similar reactions initiated by the nucleophilic attack and comprising C-N bond cleavage [29,30,31,44].

We selected sets of 500 frames for each model system from 5 ps QM/MM MD simulations of the ES complex. We estimated reactivity of ceftriaxone using Laplacian of electron density maps and Fukui electrophilicity index, *f* ^+^, of the carbonyl carbon atom. The spatial distributions of the Laplacian of the electron density were calculated in the plane of the carbonyl group of the substrate (C and O atoms) and the nucleophilic atom of Ser310, O_S_, to discriminate reactive and nonreactive moieties. Earlier works [29,30,31,44] demonstrate that this approach is a proper tool to visualize the substrate activation in nucleophilic addition reaction steps. More precisely, in nonreactive species, a carbonyl carbon atom is enveloped by the electron density concentration area. In reactive species, the carbonyl carbon atom has an electrophilic site that is an electron density deconcentration area located on the line of the nucleophilic attack. This deconcentration area is complementary to the nucleophilic site on the serine oxygen atom, being a lone electron pair oriented toward the electrophilic site. Apart from the spatially distributed Laplacian of electron density maps, an integral value of the Fukui atomic index can be calculated. The Fukui atomic index of electrophilicity, *f* ^+^, is evaluated as a difference between Hirshfeld charges [45,46] calculated for the model system with N electrons (a system under consideration) and a system with the same geometry configuration, but with an additional electron. Larger Fukui atomic electrophilicity indices correspond to the more electrophilic species. The electron density analysis was performed in the Multiwfn program package [47].

The Gibbs energy profiles for each reaction step along the reaction pathway were calculated using the umbrella sampling approach [48,49]. The sets of 5–10 ps runs were performed with harmonic potentials centered at different values of reaction coordinates. The force constant of the harmonic potential ½·K·(ξ − ξ_0_)^2^ was usually set to 40 kcal/mol/Å^2^, and additional trajectories with the K = 80–120 kcal/mol/Å^2^ in transition state regions were calculated in several runs. Harmonic potentials were centered every 0.2 Å along the reaction coordinates in regions close to minima and every 0.1 Å in transition state regions. Statistical analysis of reaction coordinate distributions was performed using both weighted histogram analysis method (WHAM) and umbrella integration (UI). The quality of distributions was monitored by their overlap and consistency of Gibbs energy profiles obtained in both WHAM and UI methods.

Classical MD simulations were carried out for 500 ns for the *apo*-form of proteins using the CHARMM36 force field for the protein and TIP3P for water molecules. Conformational diversity was analyzed using the dynamical network analysis, DNA [50]. According to this approach, a network is defined as a set of nodes connected by edges. In this work, every amino acid was represented by a single node. Any two nodes (except for the neighbors) were connected by an edge if the distance between any pair of atoms of the respective residues was less than 4 Å for more than 75% of the simulation time. Covariance and correlation matrices for dynamical network analysis were calculated with the CARMA program [51]. The MD calculations were carried out using the NAMD software package; trajectories analysis and visualization were performed in VMD program [52].

## 5. Conclusions

We propose molecular mechanisms of inhibition of PBP2 from wild-type FA19 and mutant 35/02 and H041 strains of *Neisseria Gonorrhoeae* by ceftriaxone due to the formation of the acyl–enzyme complex. In PBP2^FA19^, a reaction occurs via three elementary steps with rapid formation of the acyl–enzyme complex and slow elimination of the leaving group that is supported by X-ray data [33]. In PBP2^35/02^ and PBP2^H041^ from the mutant strains, a reaction happens in two elementary steps, as cleavage of the C–N bond in the β-lactam ring and elimination of the leaving group of the antibiotic occur simultaneously. Differences in the energy barrier of the first step of the reaction as well as subsequent stabilization of the tetrahedral intermediate are governed by the efficiency of the substrate activation in the ES complexes. It is quantified from the QM/MM MD trajectories of the ES complexes with subsequent electron-density-based analysis of features of the carbonyl carbon atom of ceftriaxone. Both Laplacian of electron density and atomic Fukui electrophilicity indices consistently demonstrate more efficient activation of ceftriaxone by PBP2^FA19^ than by PBP2 from the mutant strains. Dynamical network analysis of classical MD trajectories reveals that ceftriaxone binding affinity is higher for PBP2^FA19^. Thus, we clarify that the increase in ceftriaxone resistance of PBP2 from the mutant strains evaluated from the decrease in *k*_2_/*K_S_* [22] can be explained by both decreases in the acylation rate constant, *k*_2_, and increases in the dissociation constant, *K*_S_.

## Data Availability

Not applicable.
